# The Making of the Andrea Wave and other Rogues

**DOI:** 10.1038/srep44124

**Published:** 2017-03-08

**Authors:** Mark A. Donelan, Anne-Karin Magnusson

**Affiliations:** 1 Rosenstiel School of Marine and Atmospheric Science, University of Miami, Miami, Florida, USA; 2 Norwegian Meteorological Institute, Bergen, Norway

## Abstract

Unexpectedly large ocean waves or ‘rogues’ are sometimes claimed to be the cause of damage to ships at sea and to offshore structures. While wind-driven wave models are capable of predicting the average characteristics of waves, the maximum height of rogues that may occur is yet unknown. Rogues form in the open ocean through the addition of elemental wave trains or groups and, infrequently, with many elements coming together in phase, producing rogues. Here we perform directional analyses on one of the steepest rogues ever recorded: the Andrea wave. We find that the Andrea wave was close to the breaking-limited height. Analysis of the 72 twenty minute records on the day of the Andrea wave yields encounter return periods of about 21 days for maximally steep waves, while less steep rogues occur about twice daily. An explicit formula is given for the encounter probability, based on the target area. This work answers the critical questions regarding rogues in the design and operation of ships and offshore structures: how high can rogues be and how frequently they occur.

The Andrea rogue wave was recorded from a bridge between two Ekofisk platforms in the North Sea (56° 30′ N, 3° 12′ E) on November 9, 2007 at 00:54:22 UTC^1^. Rogue waves or their destructive effects have been reported hundreds of times but few have been measured with reliable instruments and recorded for subsequent analysis. The Andrea wave was one such and is unique in that it was measured by not one but a square (2.6 m side) array of platform-mounted surface elevation sensors (laser range-finders, accuracy of ±4 mm), “LASAR”. This enables analysis of the directional properties of constituent groups that sometimes come together to produce unusually high waves or rogues. Unusually steep waves are classified as rogues if either: (a) the height (crest from preceding trough), H is greater than twice the significant height, Hs (Hs = 4 times the standard deviation of the wave record); or (b) the crest height, Crx, above mean sea level (MSL) is greater than 1.25 times Hs^2^. Rogue waves are not necessarily huge, but they are much bigger than the surrounding waves. The Andrea wave (Fig. 1, bottom panel) was among the most roguish of all recorded rogues^1^,^3^,^4^: Crx/Hs = 1.63 and H/Hs = 2.3, and the steepest recorded with a directional array. This enables the use of the Wavelet Directional Method (WDM)^5^,^6^ to obtain frequency, wavenumber, amplitude and direction of the passing waves at each point in time.

Here we take the Andrea wave apart and examine how the constituent groups came together to produce such a steep wave. In addition we analyse the 72 twenty minute records on November 9, 2007 – a total of 13,535 waves. We ask the questions: are these waves expected from crest height statistics of weakly nonlinear random seas? could the Andrea wave have been even larger? is there a limit to how high rogues can be in a given sea-state? *i.e*. Crx/Hs < Limit? and what is the encounter probability of rogues? The directional analysis of the Andrea wave allows us to place limits on the largest single wave that could occur in the open ocean for a given significant height. The significant height is predictable by modern wind-driven wave models and so is the appropriate scaling height for rogues.

## Group analysis – unmasking the rogue

**Figure 1:**
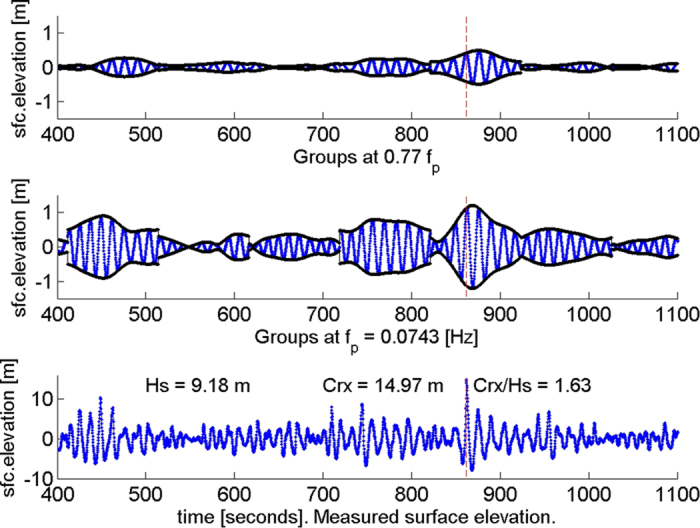
The Andrea wave (bottom panel) and two component groups centered on the spectral peak frequency, f_p_ (middle panel) and 0.77 f_p_ (top panel). The envelopes are indicated in black and the location of Andrea’s crest is marked (dashed red line) in all panels.

The standard model of the distribution of wave energy at any location considers a large collection of trains of small amplitude waves of various frequencies (or wavelengths), propagation directions and random phases as dictated in the mean by prescribed directional spectra. The ubiquitous group structure – apparent to even a casual observer and called “sets” by surfers – arises through the interference of the trains travelling in different directions at speeds that depend on wavelength. An alternate view^7^ has waves travelling in groups with constant or slowly varying wavelengths and propagation directions. This is the basis for the WDM^5^,^6^, in which the wave records are decomposed into frequency bins having time dependent amplitudes, wavelengths and directions. The resulting group structures in two of these frequency bins are shown in Fig. 1, for a section of the record that includes the Andrea wave, which occurred at 862 seconds. The surface elevation (blue) and amplitude envelope (black) are graphed at the peak (most energetic) frequency, f_p_ = 0.0743 Hz (middle panel) and at 0.77 f_p_ (top panel). The measured Andrea wave is in the bottom panel. The groupness of the components is more apparent compared to that of the wave record.

The underlying group structure of waves in each frequency band is an ubiquitous feature of wind-generated waves and supports the alternate view of waves travelling in groups rather than constant amplitude trains^5^,^7^. Unusually high waves occur when several groups come together with their crests at or near their envelopes’ maxima. This is apparent in the rogue, Andrea, as is shown in Fig. 1 for two frequency bands. (The abrupt transitions between some groups signal changes in direction of the waves.) Rogue waves have been shown to be due primarily to frequency focusing (constructive interference) with a higher order contribution from the non-resonant interaction of fundamental and bound harmonics^4^. For ocean waves, the crest enhancement due to modulational instability has been shown – theoretically^8^, numerically^9^ and observationally^10^ – to be minor. This is to be expected since the dispersive nature of gravity waves, at the root of frequency focusing, operates on a time scale of order T, the spectral peak period; whereas the modulational instability, due to quasi-resonant third order interactions, evolves much more slowly as order T/mean square slope of interacting waves^11^.

**Figure 2:**
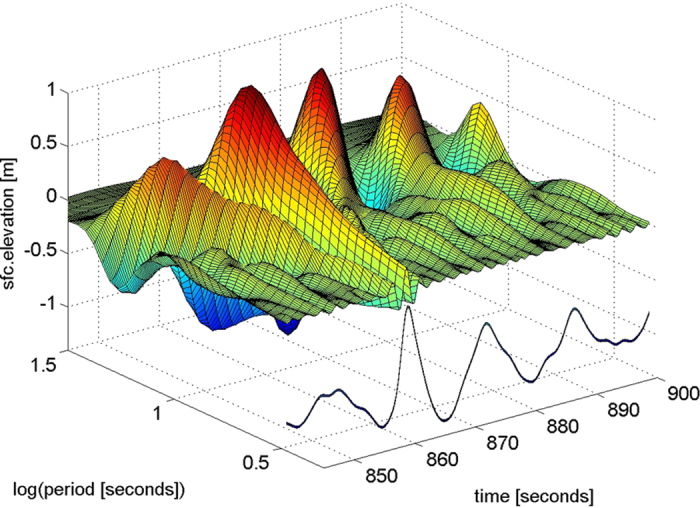
Surface elevation of groups in each period band displayed on axes of time and logarithm of period in seconds. All 32 periods are displayed and their sum (divided by 20) is graphed along the time axis. The Andrea crest is at 862 s.

The frequency focusing across the spectrum is evident in Fig. 2, where time series of the surface elevation in each group are plotted on axes of time and logarithm of period. The Andrea wave, with crest at 862 s, shows frequency focusing across the board unlike its three neighbours. Furthermore, the amplitudes of the high frequency groups are much larger at Andrea’s crest than elsewhere in the figure. This may be a reflection of the non-resonant interaction of fundamental and bound harmonics.

**Figure 3:**
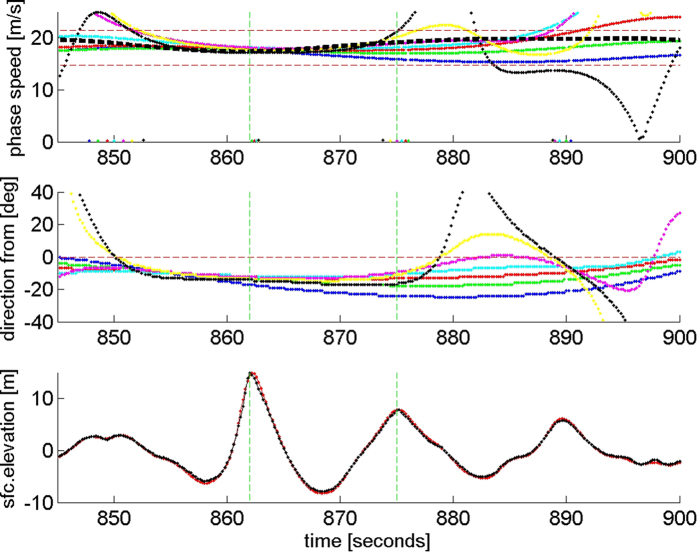
The Andrea wave measured (black) and reconstructed (red) for 55 seconds - bottom panel; the phase speeds (top panel) and directions (middle panel) of the waves in the 7 frequency bands about the peak: f/f_p_ = 0.77, 0.84, 0.92, 1, 1.09, 1.19, 1.3; color coded: blue, green, red, cyan, magenta, yellow, black. The range of theoretical phase speeds for these 7 frequencies is indicated by the thin dashed red lines. The energy (amplitude squared) weighted average (0.5f_p_ to 1.5f_p_) phase speed is indicated (top panel) by the heavy dashed black line. The time location of Andrea’s crest and the following crest are marked (dashed green line) in all panels.

In Fig. 3 we take a closer look at the make-up of the crest of the Andrea rogue (862 s) and at the following crest (875 s), which was half as high (7.5 m) – big, but not a rogue. The bottom panel displays the time series of the four waves near Andrea (black) and the reconstructed waves (red) from addition of the 32 group series logarithmically spaced from 0.03125 Hz to 0.4585 Hz. The surface elevation was sampled at 5 Hz and the (directional) analysis is limited by the array size (2.6 m square) to 0.46 Hz. The minor difference between red and black lines (bottom panel) reflects this effective low-pass filtering of the reconstructed waves. In the top and middle panels are the phase speeds and directions of the waves in the 7 frequency bands about the peak: f/f_p_ = 0.77, 0.84, 0.92, 1, 1.09, 1.19, 1.3; color coded: blue, green, red, cyan, magenta, yellow, black. The wavelet directional analysis yields the wavenumber (2*π*/wavelength), amplitude and frequency of each component group at every sampling time, thereby enabling the calculation of instantaneous phase speeds. The energy (amplitude squared) weighted average (0.5f_p_ to 1.5f_p_) phase speed is indicated (top panel) by the heavy dashed black line, which is 17.85 m/s at Andrea’s crest. During the passage of Andrea these large groups propagated at nearly the same direction (middle panel) and phase speed, even though the linear theoretical phase speeds varied from 21.4 m/s to 14.8 m/s (indicated by thin dashed red lines) across the groups. The second harmonic (not shown here) also travels at the peak phase speed at and near Andrea’s crest – it is indeed bound. Evidently the Andrea wave propagated for at least 8 seconds (over a distance of about 140 m) essentially without change of form: a veritable ‘wall of water’ 20.8 m, 68 ft (trough to crest) high and advancing at 17.85 m/s, 40 mph. The rapid modifications of wavenumbers in the vicinity of the peak are evidently caused by second order self interactions associated with the skewness of the surface^11^.

Rogue waves are sometimes reported as having foam-capped crests as in a spilling breaker. Spilling of the crest occurs when the horizontal orbital velocity at the crest exceeds the phase speed (or crest speed). The Andrea wave had pronounced horizontal asymmetry with a steep forward face suggesting that it was at or near spilling. The superposition method^12^ is used to calculate the theoretical horizontal orbital velocity near the crest assuming an average directional spread of 26 degrees. The average velocity in the top 50 cm exceeds the crest speed by 2 m/s *–* Andrea was capped by a spilling layer.

The recorded Andrea wave crested at 14.97 m above MSL, but it may have been even higher nearby in space and time. To explore this we evolve the groups linearly (assuming long-crestedness^3^) with their measured amplitudes, phases, frequencies and wavenumbers, over ±300 m in both directions and ±15 seconds. The reconstructed Andrea wave crested at 15.33 m, 6 m North, 14 m West and 0.2 seconds before it crossed the Ekofisk array. During the evolution of Andrea the surface elevation contour defining a rogue (1.25 Hs) was a straight line at the front 100 m long and nearly normal to the propagation direction – a ‘wall of water’ 100 m wide or about half a wavelength.

**Figure 4:**
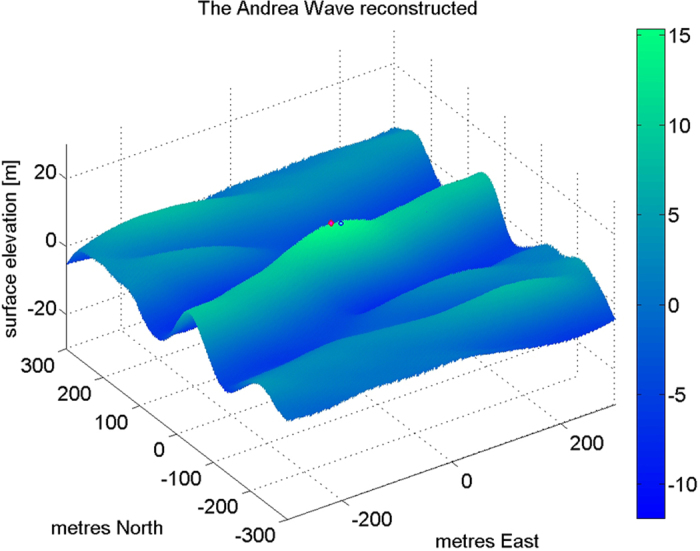
Aerial view from southwest (elevation 45 degrees) of the reconstructed Andrea wave. The colorbar indicates surface elevation. Vertical exaggeration is seven. The blue dot (location: 0,0) indicates the observed crest of 14.97 m above MSL; the red dot indicates the reconstructed maximum crest height of 15.33 m at location: 14 m West, 6 m North. The crest was advancing to the SSE (164 degrees) at 17.85 m/s.

**Figure 5:**
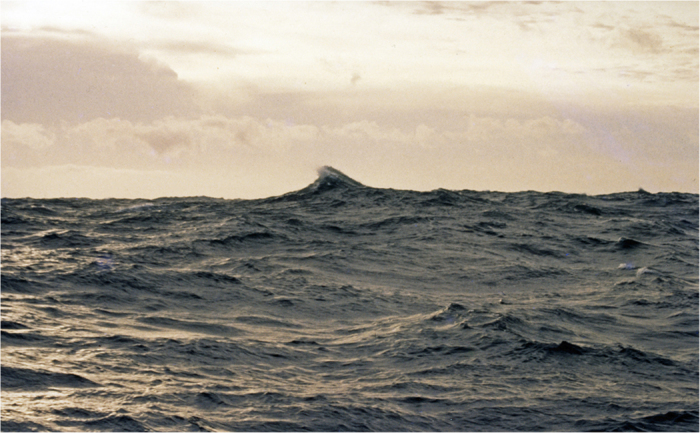
Rogue wave photograph taken from the deck of the RV Cape Henlopen in the western North Atlantic during ARSLOE (Atlantic Remote Sensing Land/Ocean Experiment). The crest of the rogue propagates from right to left. (Photograph by H. Mitsuyasu^13^).

A ‘snapshot’ of the reconstructed surface, at the time of Andrea’s recorded crest, is shown in Fig. 4. In Fig. 5 a rare photograph^13^ of an open ocean rogue wave is shown. The photograph was taken by the distinguished Japanese oceanographer Hisashi Mitsuyasu from the deck of the RV Cape Henlopen in the western North Atlantic during ARSLOE (Atlantic Remote Sensing Land/Ocean Experiment). The significant height (measured by a nearby accelerometer buoy^13^) was 4 m and the height (trough to crest) of the rogue was roughly estimated visually at 10 m – a very roguish wave indeed: H/Hs = 2.5 compared with Andrea’s H/Hs = 2.3. Both reconstructed Andrea and the photographed rogue show the essential loneliness of rogues – a single very high wave in a general background of much smaller waves; both appear to be spilling at the top of a very steep forward face and may be limited in height by breaking.

### How steep can rogues be? – optimizing Andrea

**Figure 6:**
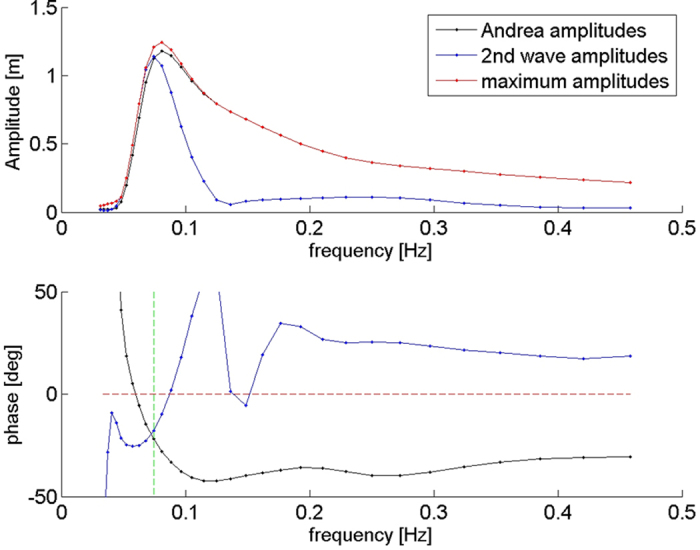
The amplitudes (top) and phases (bottom) of the 32 frequency bands at the crest of the Andrea wave (black), the crest of the following (2nd) wave (blue), and the maximum at each frequency (red). The dashed green line marks the peak frequency.

The Andrea wave is among the steepest rogues ever recorded and it was spilling at the crest. The steepness of the wave at the time of maximum crest, defined by the product of crest height (14.97 m) and the measured peak wavenumber at the crest (0.0282 radians/m), Crx · k_p_, was 0.422. The reconstructed Andrea reaches a crest height of 15.33 m, i.e. steepness of 0.432. Could the Andrea wave have been even larger had the groups been combined with their maximum amplitudes in phase across the spectrum? Figure 6 shows the distribution of group amplitudes and phases at the crest for the measured Andrea wave (black), the wave immediately following (blue) and the maximally steep wave (red) that could have occurred somewhere in the vicinity of Ekofisk during the 20 minutes of the Andrea record. The phases of the Andrea wave groups vary smoothly within ±45 degrees, suggesting interaction among the groups. The Andrea wave group amplitudes were optimum except near the peak frequency, where they are some 5% low. By contrast, the following wave group amplitudes are low above the peak frequency. Consequently the following wave was only half as high as Andrea. The maximally steep rogue could have been 16.59 m high, steepness 0.468. Such a wave would have been intensely breaking and consequently limited in height.

In a comprehensive set of laboratory experiments leading to his Ph.D. thesis, Michael Allis explored the breaking limited steepness of focused groups^14^. For directionally narrow focused groups he found that the breaking-limited steepness was 0.44. As we have seen, Andrea at steepness 0.422 was breaking (spilling) and therefore close to being limited by intense breaking. At steepness of 0.44 Andrea would have had a crest height of 15.61 m and Crx/Hs (crest height/significant height) of 1.7. The corresponding normalized height limit (height/significant height) is 2.4. These two numbers define the largest rogues that may occur in any open ocean storm. The wavelength, 
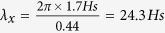
, and the central frequency may be determined from the dispersion relation. For worst case design and operation of ships and offshore structures, heights, wavelength and frequency are sufficient to constrain the breaking-limited rogues that might occur in any storm. The significant height may be forecasted by any of several existing spectral models.

### How rare are rogues?

Rogue waves are isolated events probably occurring several times during storms at various locations. Attempts to find the statistics of rogues with point measurements lead to the conclusion that they are indeed rare. However, if we could image continuously the entire storm-excited water surface, we would undoubtedly find that their rarity has been greatly exaggerated^3^,^15^. Here we examine the 72 twenty minute records at Ekofisk on November 9, 2007 (Day 9). The wind speed was quite steady at 22 m/s in the first 7 hours and thereafter decreased steadily to 9 m/s at the end of the day^1^. Correspondingly Hs increased from 9 m to 10.5 m and then decreased to 4 m as the wind speed fell.

**Figure 7:**
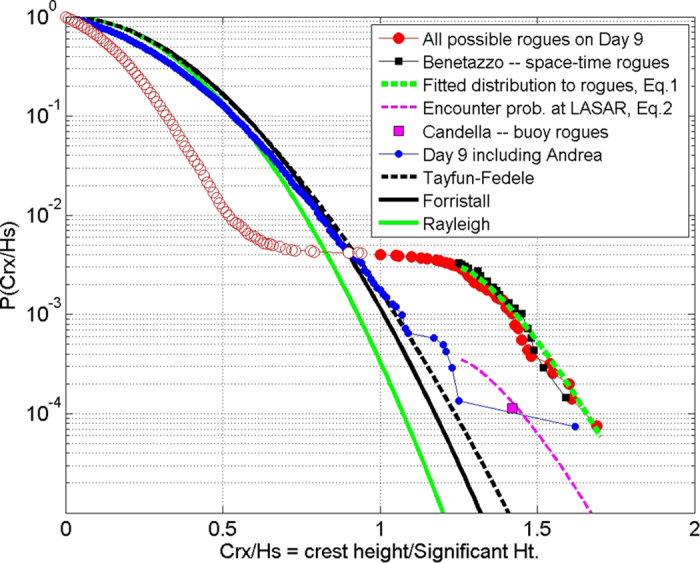
The probability of exceedance and encounter probability of normalized crest heights (Crx/Hs) for various populations of waves as indicated in the legend. The red dots above Crx/Hs = 1 represent the possible rogues on Day 9. The open red circles are an artifact of combining all rogue time series together; such distributions are not observable.

The probability of exceedance of normalized crest heights (Crx/Hs) is graphed (Fig. 7) for various populations of waves – observed and simulated to optimize crest heights. Analysis of Day 9 (24 hours) reveals Andrea was the only very steep rogue (Crx/Hs = 1.63); one other was at the defining rogue limit (Crx/Hs = 1.25). The linear (Rayleigh), second order (Forristall^16^) and third order (Tayfun-Fedele^4^) exceedance probability distributions are shown by the green, black and dashed black curves respectively. The observed exceedance probability for day 9 (blue dots) closely matches the Tayfun-Fedele distribution in the range 0.8 < Crx/Hs < 1.08. Beyond this the observed exceedance probability abruptly exceeds the Tayfun-Fedele distribution. These are the “unexpected” high waves or rogues. This clearly indicates that the standard model of small amplitude trains summed in random phase, on which the Rayleigh, Forristall and Tayfun-Fedele distributions^4^,^16^ are based, is not viable for rogues. Using instead the constructive interference of groups as the model, the highest waves that might have been formed elsewhere in the vicinity of the measuring array (LASAR) are estimated by shifting the observed amplitudes and phases (at each frequency) to produce complete focusing of the groups’ maximum envelopes at the time of the highest observed wave in each record. The resulting exceedance probability distribution for these possible rogues on Day 9 is indicated by the connected red dots. The presence of rogues in the record means that there are fewer intermediate size waves as can be seen by comparing the Day 9 observed distribution (blue dots) with the Tayfun-Fedele distribution for Crx/Hs < 0.9. This effect is amplified when there are numerous rogues (red open circles), and is an artifact of combining all rogue time series together; such distributions are not observable. The exceedance probability distribution for “all possible rogues on Day 9” (red dots) represents the marginal (time) distribution of the space-time distribution of the rogues (Crx/Hs >= 1.25).

Recent stereo-photogrammetric measurements^3^, from a tower in the Adriatic Sea, provide the first direct estimates of the space-time distribution of rogue waves. The image pairs, grabbed at 15 Hz, covered a surface area equivalent to 14.57 waves. The record duration was 1798 s or 473 waves, and 23 rogues (Crx/Hs ≥ 1.25) were captured. These yield the marginal (time) exceedance probability distribution 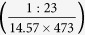 indicated with black squares. The close agreement with the probability of all possible rogues at Ekofisk on Day 9 (red dots) confirms the idea that rogues are indeed produced by constructive interference of the component groups. The dashed green curve, fitted to the two marginal distributions of rogues (red dots and black squares), is the generic exceedance probability of rogues, P_R_(Crx/Hs) given by:


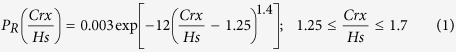


The question of whether such steep waves are predictable from the extremes of Gaussian fields has been answered^17^. Socquet-Juglard *et al*.^17^ analyzed the extremes of crest heights using Piterbarg’s theorem^18^ applied to 6 hours of 10 second period simulated waves on an area 100 × 100 km. The mean wavelength and crest length were 200 m and 750 m respectively. The most probable maximum crest height over space and time was 1.68 Hs.

Of course, the encounter probability^15^ is that which is discussed when we consider how rare rogues are. This can be obtained, for a given rogue wavelength, λ, by multiplying P_R_ by the ratio: (A_c_ + A_o_)/A_w_; where A_c_ is the area swept by a crest = α λ^2^; A_o_ is the waterline area of the target encountered; A_w_ is the area of a wave = β λ^2^. The Adriatic Sea measurements^3^ demonstrate that wind generated seas are slightly long-crested: crest length/wavelength = 1.074 on average. Therefore β = 1.074 and α can be determined by comparing the observed probabilities (blue dots at Crx/Hs = 1.17, 1.20, 1.21, 1.23) with the corresponding probabilities of the possible rogues (red dots). The encounter probabilities are 7.91 ± 2 times lower, which yields α = 0.136 and the encounter exceedance probability, EP_R_ is given by:


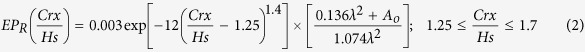


This is plotted (dashed magenta curve) on Fig. 7 for λ = 223 m and A_o_ = 6.76 m^2^, the area of the Ekofisk array.

In a year-long rogue hunt at three buoys off the Brazilian coast^19^, 442 rogues (H/Hs ≥ 2) were found in a population of 3.9 million waves. This gives an encounter probability of 1.13 × 10^−4^, which is indicated at the equivalent Crx/Hs (2 × 1.7/2.4 = 1.42) by a magenta square. The close agreement with the dashed magenta curve validates Eq. (2).

From Eq. (2), the encounter probability of rogues (Crx/Hs ≥ 1.25) is 1 in 2637 waves, which for 13.5 s period waves (like Andrea) yields a return period of about 10 hours or a 3.3% chance of finding a rogue in a 20 minute record. Maximally steep rogues (Crx/Hs = 1.7) are much more rare: the encounter probability, EP_R_(1.7) is 1 in 133,361 waves or return period of 500 hours. Very large targets (*e.g.* ships or offshore platforms) experience shorter return periods as quantified in Eq. (2).

## Discussion

While maritime engineering standards warn users about rogue (or “freak”) waves^20^, they give no guidance on the maximum heights rogues can reach or how frequently they occur. On the basis of these analyses, on the day of the Andrea storm in the North Sea, we characterize the breaking-limited rogue by: 1) crest height/significant height = 1.7; 2) trough-to-crest height/significant height = 2.4; 3) wavelength/significant height = 24.3. We find that the encounter probability of rogues (Crx/Hs ≥ 1.25) at a point is about 1 in 2637, while that for maximally steep and breaking rogues is about 1 in 133,361. The corresponding return periods for 13.5 s period rogues are 10 hours and 500 hours respectively. Larger targets experience shorter return periods. A general formula for encounter probability is provided and verified with a large data base from the South Atlantic Ocean.

## Additional Information

**How to cite this article**: Donelan, M. A. and Magnusson, A.-K. The Making of the Andrea Wave and other Rogues. *Sci. Rep.* **7**, 44124; doi: 10.1038/srep44124 (2017).

**Publisher's note:** Springer Nature remains neutral with regard to jurisdictional claims in published maps and institutional affiliations.

## References

[b1] Magnusson A. K. & Donelan M. A. The Andrea wave – characteristics of a measured North Sea rogue wave. Journal of Offshore Mechanics and Artic Engineering 135, 1–10, doi: 10.1115/1.4023800 (2013).

[b2] Dysthe K., Krogstad H. E. & Muller P. Oceanic rogue waves, Annu. Rev. Fluid Mech. 40, 287–310 (2008).

[b3] Benetazzo A. . Observation of extreme sea waves in a space-time ensemble. J. Phys. Oceanogr. 45, 2261–2275, doi: 10.1175/JPO-D-15-0017.1 (2015).

[b4] Fedele F. . Real world ocean rogue waves explained without the modulational instability. Sci. Rep. 6, 27715, doi: 10.1038/srep27715 (2016). 27323897 PMC4914928

[b5] Donelan M. A., Drennan W. M. & Magnusson A. K. Nonstationary analysis of the directional properties of propagating waves. J. Phys. Oceanogr. 26, 1901–1914 (1996).

[b6] Donelan M., Babanin A., Sanina E. & Chalikov D. A comparison of methods for estimating directional spectra of surface waves. J. Geophys. Res. Oceans 120, 5040–5053, doi: 10.1002/2015JC010808 (2015).

[b7] Mollo-Christensen E. & Ramamonjiarisoa A. Modeling the presence of wave groups in a random wave field. J. Geophys. Res. 83, 4117–4122 (1978).

[b8] Fedele F. On the kurtosis of ocean waves in deep water. J. Fluid Mech. 782, 25–36 (2015).

[b9] Adcock T. A. A., Taylor P. H. & Draper S. Nonlinear dynamics of wave-groups in random seas: unexpected walls of water in the open ocean. Proc. R. Soc. A 471, 20150660 (2015).

[b10] Christou M. & Ewans K. Field measurements of rogue water waves. J. Phys. Oceanogr. 44, 2317–2335 (2014).

[b11] Phillips O. M. The Dynamics of the Upper Ocean. Cambridge Univ. Press, Cambridge, U.K., 336 pages (1977).

[b12] Donelan M. A., Anctil F. & Doering J. C. A simple method for calculating the velocity field beneath irregular waves. Coastal Engineering 16, 399–424 (1992).

[b13] Mitsuyasu H. Looking Closely at Ocean Waves: From Their Birth to Death (TERRAPUB, 2009).

[b14] Allis M. J. The Speed, Breaking Onset and Energy Dissipation of 3D Deep-Water Waves. *The University of New South Wales*, PhD thesis, 340 pages (2013).

[b15] Cavaleri L. . The Draupner wave: A fresh look and the emerging view. J. Geophys. Res. Oceans, 6061–6075, doi: 10.1002/2016JC011649 (2016).

[b16] Forristall G. Z. Wave crest distributions: observations and second-order theory. J. Phys. Oceanogr. 30, 1931–1943 (2000).

[b17] Socquet-Juglard H. . Spatial extremes, shapes of large waves, and Lagrangian models. *Proceedings of the Rogue Waves 2004 Workshop*, M. Olagnon and M. Prevosto, eds, Brest, France, Oct. 20–22, 11 pages (2004).

[b18] Piterbarg V. I. *Asymptotic methods in the theory of Gaussian processes and fields*, AMS Transl. of Math. Monographs, **148**, Providence, R. I. (1996).

[b19] Candella R. N. Rogue waves off the south/southeastern Brazilian coast. Nat. Hazards, doi: 10.1007/s11069-016-2312-2 (2016)

[b20] Bitner-Gregersen E. M. & Gramstad O. Rogue waves. Position paper 05-2015. DNV-GL Strategic research & Innovation. 60 pages (2016).

